# Effects of Peer- or Professional-Led Support in Enhancing Adherence to Wearable Monitoring Devices Among Community-Dwelling Older Adults: Systematic Review of Randomized Controlled Trials

**DOI:** 10.2196/53607

**Published:** 2024-06-20

**Authors:** Colette Sze Wing Chan, Mandy Ming Pui Kan, Arkers Kwan Ching Wong

**Affiliations:** 1 School of Nursing The Hong Kong Polytechnic University Hung Hom China (Hong Kong)

**Keywords:** wearable monitoring device, older adults, adherence, systematic review, healthy aging, peer support, professional help, support, peers, peer, professionals, wearable, monitoring devices, monitoring device, community-dwelling, older adults, older adult, aging, aging, elderly

## Abstract

**Background:**

Despite the well-documented health benefits associated with wearable monitoring devices (WMDs), adherence among community-dwelling older adults remains low. By providing guidance on the purpose and benefits of using WMDs, facilitating goal-setting aligned with the device’s features, promoting comprehension of the health data captured by the device, and assisting in overcoming technological challenges, peers and health care professionals can potentially enhance older adults’ adherence to WMDs. However, the effectiveness of such support mechanisms in promoting adherence to WMDs among older adults remains poorly understood.

**Objective:**

The aims of this systematic review were to examine the effects of peer- or professional-led intervention programs designed to improve adherence to WMDs among community-dwelling older adults and to identify the intervention components that may positively influence the effects of the intervention.

**Methods:**

We conducted a comprehensive search across 7 electronic databases (Cochrane Central Register of Controlled Trials [CENTRAL], PubMed, EMBASE, PsycINFO, British Nursing Index, Web of Science, and CINAHL) to identify articles published between January 1, 2010, and June 26, 2023. We specifically targeted randomized controlled trials that examined the impact of peer- or professional-led interventions on enhancing adherence to WMDs among individuals aged 60 years and older residing in the community. Two independent reviewers extracted data from the included studies and assessed the potential risk of bias in accordance with the Cochrane Risk of Bias tool for randomized trials, version 2.

**Results:**

A total of 10,511 studies were identified through the database search. Eventually, we included 3 randomized controlled trials involving 154 community-dwelling older adults. The participants had a mean age of 65 years. Our review revealed that increasing awareness of being monitored and implementing the SystemCHANGE approach, a habit change tool focusing on personal goals and feedback, were effective strategies for enhancing adherence to WMDs among older adults. All of the included studies exhibited a low risk of bias.

**Conclusions:**

By collaboratively designing specific goals related to WMDs with health care professionals, including nurses and physicians, older adults exhibited a higher likelihood of adhering to the prescribed use of WMDs. These goal-setting tools provided a framework for structure and motivation, facilitating the seamless integration of WMDs into their daily routines. Researchers should prioritize interventions that target awareness and goal-setting as effective approaches to enhance adherence to WMDs among older adults, thereby maximizing the realization of associated health benefits.

## Introduction

### Overview

The global population is ageing at an unprecedented speed, with the number of people older than 65 years expected to double from 1 billion in 2020 to 2.1 billion in 2050 [1]. The World Health Organization has transformed this challenge into an opportunity by putting forward the concept of “healthy ageing,” which is about creating an environment that enables older adults to age with good health and well-being [2]. However, to put this concept into practice, older adults, who are usually living with multiple chronic health conditions, have to be competent in carrying out self-management activities, such as medication management, health monitoring, and fall prevention. This is more easily said than done, although it is not impossible with the help of emerging digital self-management technologies.

Wearable monitoring devices (WMDs) are a common digital technology specifically designed to help people take charge of their own health and develop an individualized care or treatment plan. WMDs, which include but are not limited to activity trackers, pedometers, and smartwatches, are useful for collecting a wide range of personal health data in real time, such as blood pressure and heart rate, and revealing an individual’s physical movements, GPS location, and calorie expenditure [3]. Not only can WMDs ensure the safety of users by the early screening of symptoms, but they can also empower users to continuously measure their health and well-being independently without the help of family members or health care professionals. Previous studies have demonstrated that WMDs are particularly effective for older adults with chronic diseases. A systematic review of the effectiveness of WMDs reported that WMDs could bring about a noticeable increase in daily step count and moderate-to-vigorous physical activities, although its impact on reducing sedentary behavior was found to be insignificant [4].

Despite the benefits provided by WMDs, studies suggested that the rate of adherence to WMDs among older adults is low. A study showed that 1 in 3 adults abandoned the use of WMDs within 6 months after purchasing one, while those aged 70 years and older discontinued its use after only 2 weeks [5]. Nonadherence to WMDs affects the completeness of health data and impairs early detection of the aggravation of a disease, which will eventually have a negative impact on the health of users [6]. Previous studies indicated that the characteristics of a WMD, such as its ease of use, size, security and privacy setting, and maintenance cost, as well as the characteristics of older adults, such as their technological competence and perception of the usefulness of the WMD, are some factors that may influence the intention of users to keep using the WMD [5,7-9].

Peers or health care professionals are crucial in promoting the long-term use of WMDs among older adults [10-12]. The literature suggests that these people can help older adults gain insights into the purpose of using the WMD, facilitate the planning and setting of goals with regard to the features of the WMD, understand the health data captured in the WMD, and overcome technological difficulties [11,13,14]. However, to the best of our knowledge, there has been no systematic review evaluating the effects of these peer- or professional-led intervention programs on improving long-term adherence to WMDs among older adults or identifying the specific components in these programs that can lead to positive effects. Conducting such reviews could help researchers and policy makers design targeted strategies to promote the use of WMDs, ultimately leading to better health monitoring and improved health care outcomes.

### Objectives

This review aims to explore the effects of peer- or professional-led intervention programs on enhancing adherence to WMDs among community-dwelling older adults and to disentangle the following research questions:

1. What existing interventions have been designed to improve adherence to WMDs among community-dwelling older adults?
2. What are the effects of these interventions on adherence to WMDs among community-dwelling older adults?
3. What intervention components may positively influence the effects of interventions aimed at enhancing adherence to WMDs?

## Methods

### Database Selection and Search Strategy

This review was conducted according to the PRISMA (Preferred Reporting Items for Systematic Reviews and Meta-Analyses) [15] guidelines, and the protocol was registered on PROSPERO (CRD42023395459).

A total of 7 electronic databases (Cochrane Central Register of Controlled Trials [CENTRAL], PubMed, EMBASE, PsycINFO, British Nursing Index, Web of Science, and CINAHL) were searched to identify articles published between January 1, 2010, and June 26, 2023. A combination of search strings related to “wearable monitoring devices,” “older adults,” “intervention program,” and “adherence” were used. A complete search strategy for each database is shown in Multimedia Appendix 1. A manual search of the reference lists of the included articles was performed to identify additional relevant articles.

### Study Selection

This review intended to summarize evidence regarding the effectiveness of peer- or professional-led interventions on improving adherence to WMDs among community-dwelling older adults by comparing those who did and did not receive the intervention. For this review, our definition of peer-led and professional-led interventions referred to how peers and health care professionals contributed to promoting adherence to WMDs. The eligibility criteria were developed using the PICO (population, intervention, comparison, and outcomes) process.

### Inclusion and Exclusion Criteria

Articles on studies that adopted a parallel randomized controlled trial (RCT), cluster RCT, or crossover RCT were eligible for inclusion in the current review if the study participants were community-dwelling older adults (with or without chronic diseases) aged ≥60 years using a WMD (population); a peer-led or professional-led intervention that sought to improve WMD adherence was tested (intervention); a comparison was made with participants not exposed to the interventions (comparison); and their adherence to the WMD was measured (outcomes). Meanwhile, articles were excluded if they were in a language other than English or Chinese; involved institutionalized or hospitalized patients; involved patients who were cognitively impaired or physically limited, making them incapable of adhering to the WMD; and focused mainly on the clinical outcomes of using WMD.

### Screening Process

Articles identified from databases were imported to EndNote 20 to remove duplicates. All titles and abstracts were first independently screened by 2 reviewers (MMPK and CSWC) based on the eligibility criteria to identify potential articles. The 2 reviewers then independently reviewed the full text of these articles to determine whether the articles satisfied the selection criteria. Disagreements were resolved by consensus with a third reviewer (AKCW).

### Data Extraction and Appraisal of the Methodological Quality

The 2 independent reviewers extracted data from the included studies to a Microsoft Excel template sheet and assessed the potential risk of bias in the studies, in accordance with the Cochrane Risk of Bias tool version 2 (RoB 2) for randomized trials [16]. The corresponding authors of the included articles were contacted for missing data or clarification, if necessary.

The data extracted from each eligible article included the following study characteristics:

- Methods: country and periods during which the study was conducted, study design, and dropout rate
- Participants: characteristics of all the participants by groups (eg, the number of participants, their mean age, gender, ethnicity, educational level, and socioeconomic status)
- Features of the WMDs
- Interventions: description of the intervention type, provider, content, frequency, and duration
- Controls
- Outcomes: adherence, sustained usage, and time points

The RoB 2 tool addressed the biases that potentially affect the results of an RCT in five domains: (1) bias arising from the randomization process; (2) bias due to deviations from intended interventions; (3) bias due to missing outcome data; (4) bias in measuring the outcome; and (5) bias in selecting the reported result. The level of bias in each domain and the overall risk of a study were judged to be either a “low risk of bias,” “some concerns,” or a “high risk of bias” [16].

### Data Synthesis

We have included qualitative studies in our research and presented a summary of the findings in a narrative format. We conducted both a thematic synthesis, following the narrative synthesis guidelines established by Popay et al [17], and a more theory-driven analysis to interpret the results.

## Results

### Selection

**Figure 1:**
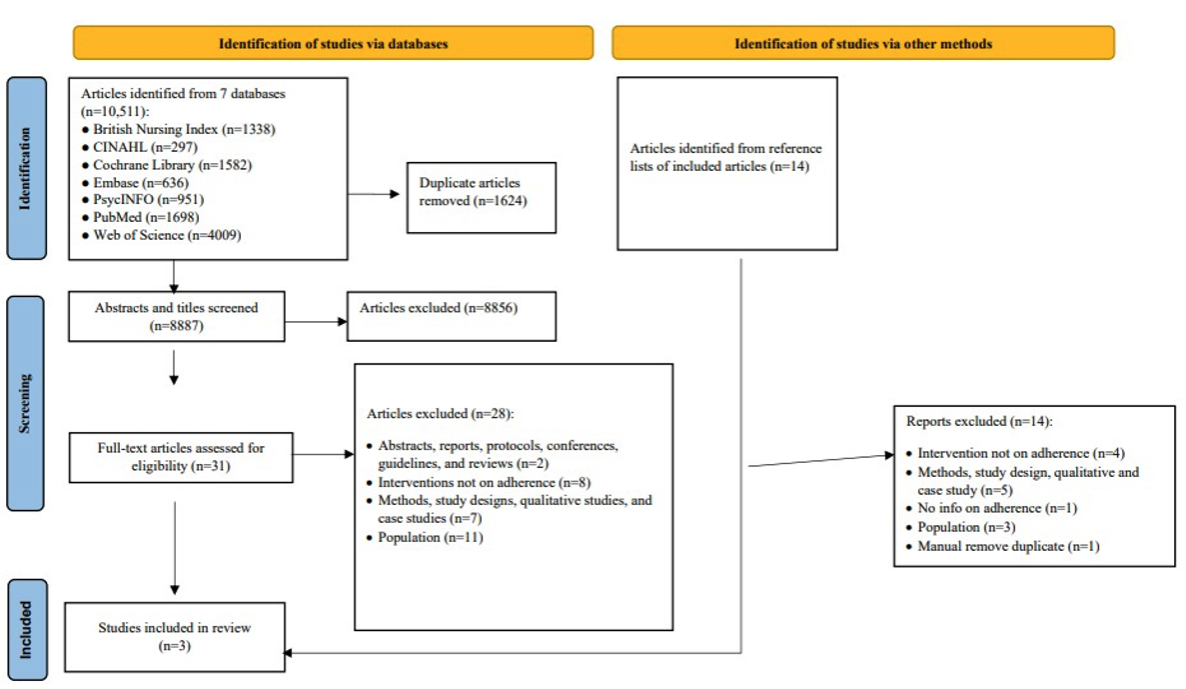
The PRISMA (Preferred Reporting Items for Systematic Reviews and Meta-Analyses) flow diagram.

A total of 10,511 studies were identified from searching the databases. After removing 1624 duplicated studies, 8887 articles were screened based on their titles and abstracts. The full texts of 33 articles that were deemed eligible were then assessed. Finally, 3 articles were selected for the current review. No further articles were identified from the reference list of the included articles. The complete study selection process is illustrated in Figure 1.

### Study Characteristics

Of the 3 selected studies, 2 were conducted in the United States [18,19], and 1 was conducted in the Netherlands [20]. The study with the highest dropout rate (75.4%) was that by Lutjeboer et al [20]. All 3 articles used a 2-arm parallel-group RCT design [18-20].

Table 1 summarizes the 3 articles that were included in this study, which involved a total of 154 participants. The mean age of the participants was 65 years. The percentage of female participants ranged from 41.7% to 61.8%. Two studies focused on older adults who had undergone a kidney transplant [18,19], while the remaining study involved older adults with no major health problems [20]. The older adults in the included studies had a relatively high level of education, with more than half possessing an associate degree or higher [18,19]. The majority of participants in the two studies had an annual household income of more than US $20,000 [18,19]. This suggests that, in general, the participants had a fair to high socioeconomic status.

**Table 1:** Summary of the included articles (N=3).

Author; year; country	Design	Sample size, n	Dropout rate (%)	Mean age (year); female participants (%)	Study population; ethnicity	Education level	Information about the WMD^a^	Randomized ratio	Intervention arm 1	Comparison arm (intervention arm 2)	Outcomes measure and time points	Results	Intervention component
Lutjeboer et al (2020) [20]; Netherlands	Parallel RCT^b^	55	75.4	65.9; 1.8	Adults who received their first pair of orthopedic footwear for a health condition; NS^c^	NS	Custom-made orthopedic footwear with a temperature sensor to measure use and nonuse of orthopedic footwear	1:1	Aware of the temperature on the sensor that measures use and nonuse (n=25)	Only knew the shoe temperature was being measured (n=30)	Adherence to orthopedic footwear reflected by wearing time in weeks 6 to 12	Intervention group: 7.32 hours/day; control group: 6.11 hours/day	Conscious awareness of WMD adherence being monitored enhances adherence to WMD
O’Brien et al (2020) [19]; United States	Parallel RCT	53	11.7	65.4; 41.7	Older kidney transplant recipients; 98% non-Hispanic	58% of participants had an associate degree or higher; over 90% had an income of > US $20,000	Fitbit Charge 2 activity tracker measuring daily steps	1:1	Multicomponent physical activity intervention SystemCHANGE (ie, personal system-based solutions support with visual feedback from the activity tracker; n=27)	Standard educational information about posttransplant (n=26)	Adherence to wearing the activity tracker at 6 months	Intervention group: 100%; control group: 92.3%	Tailored individual activity goals with visual feedback from WMD promotes adherence to WMD
O’Brien et al (2021) [18]; United States	Parallel RCT	46	23.3	65.4; 41.7	Older kidney transplant recipients; 87% non-Hispanic	52% of participants had an associate degree or higher; 77% had an income of >US $20,000	Fitbit Charge 2 activity tracker measuring daily steps	1:1	Multicomponent physical activity intervention SystemCHANGE (ie, personal system-based solutions support with visual feedback from the activity tracker; n=23)	Standard educational information about posttransplant healthy lifestyle choices (n=23)	Adherence to wearing the activity tracker at 12 months	Intervention group: 96.5%; control group: 80.8%; odds ratio 6.6 (95% CI 2.1-21.2)	Tailored individual activity goals with visual feedback from WMD promotes adherence to WMD

^a^WMD: wearable monitoring device.

^b^RCT: randomized controlled trial.

^c^NS: not specified.

### WMD Information and Adherence Outcomes

The two studies that involved Fitbit smartwatches stated the rate of adherence to WMDs as their outcome but did not specify how it was measured [18,19]. In the third study, which involved orthopedic footwear for people with a foot condition, adherence (both use and nonuse) was measured using a built-in temperature sensor [20].

### Objective 1: Existing Interventions to Improve WMD Adherence

In the 3 studies, we identified 2 interventions aimed at enhancing adherence to WMDs. In the study conducted by Lutjeboer et al [20], the intervention used was awareness of being monitored for wearing time [20]. Specifically, the “no awareness” group only knew that their footwear contained WMDs, which in this case were temperature sensors, but did not know the purpose of the study. On the other hand, the “awareness” group received a letter explaining the purpose of the study, thus ensuring they were well informed of being monitored on the time they spent wearing the WMDs.

Another intervention was the SystemCHANGE approach, in which the focus was on changing habits by targeting personal goals and providing feedback through 4 steps [18,19]. The first step involved identifying individuals who had an impact on the participants’ level of physical activity. These individuals, recognized by the participants as significant figures, could include peers, family members, and health care professionals. Then, the participants were asked to recognize their routine and the impact of that routine. Subsequently, the identified routine was graphically represented to help the participants understand how it affected their physical activity patterns. The final step entailed developing a customized solution for the participants, with the assistance of WMDs.

### Objective 2: Effects of Interventions on WMD Adherence

Although the interventions in all 3 studies were not specifically designed to promote adherence or evaluate the components contributing to adherence, they did report adherence rates at single time points. The duration of the adherence that was measured varied across the studies, ranging from 12 weeks [20] to 12 months [18]. The results demonstrated that the participants’ awareness of being monitored while using WMDs was effective in enhancing adherence. This was evident from the longer mean wearing time in the intervention group (7.32 hours/day) compared to the comparison group (6.11 hours/day) [20]. Similarly, the studies found that participants were more likely to adhere to the WMDs when using the SystemCHANGE approach, a habit change tool that targets personal goals and provides feedback [18,19]. Adherence among the intervention participants in these 2 studies was 100% at 6 months and 96.5% at 12 months, in contrast to 92.3% and 80.8%, respectively, for the control participants.

### Objective 3: Components That Positively Influence the Intervention Effect

In Lutjeboer et al’s study [20], physicians played a crucial role in promoting adherence to WMDs. The effectiveness of the intervention relied on physicians providing information about the purpose of the study to the participants. In the studies conducted by O’Brien et al [18,19], both nurses and participants worked together to achieve the intervention effect. The nurses were responsible for guiding the participants to reflect on their lifestyle during the monthly sessions, using the SystemCHANGE approach. Meanwhile, the participants actively engaged in formulating and implementing the action plan, taking an active role in the intervention process.

### Risk of Bias Assessment

**Figure 2:**
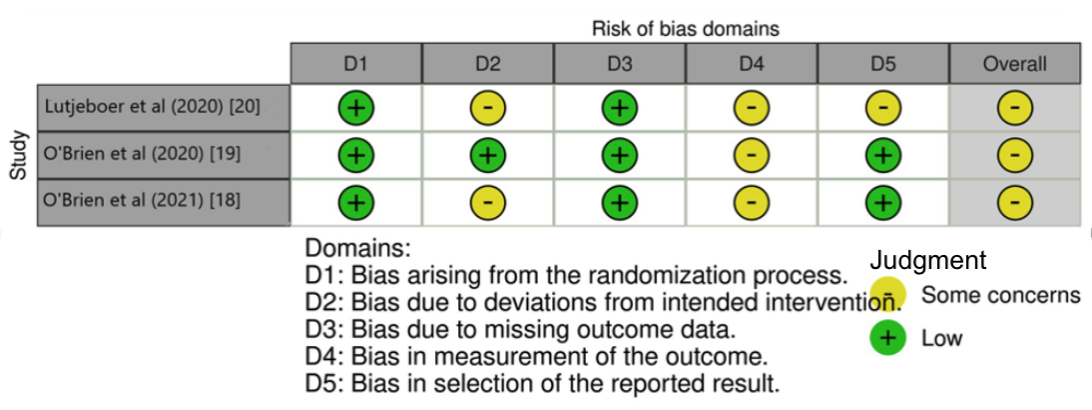
Risk of bias of the included articles (N=3).

Figure 2 [21] indicates that in the RoB 2 bias assessment, there were “some concerns” about the 3 articles [18-20]. All of the included studies [18-20] demonstrated a low risk of bias in the domains of “randomization process” and “outcome measurements.” In 2 studies [18,20], there was some concern regarding deviation from intended interventions, as the research staff may have been aware of the intervention during the trial. The remaining study was rated as being of “low risk” for this issue [19]. In terms of the measurement of the outcome, there were some concerns with all 3 articles [18-20], as knowledge of the intervention could have affected the study outcomes. Regarding the selection of the reported result, there were “some concerns” in 1 article [19], while the other 2 were considered as being of “low risk” [18,19].

## Discussion

### Principal Findings

In summary, this systematic review identified 3 RCTs, published between 2020 and 2021, that reported adherence to WMDs. Our findings revealed that low-cost interventions aimed at increasing adherence to WMDs were effective. Specifically, the review highlighted 2 types of interventions that showed promise in enhancing adherence to WMDs among older adults. The first intervention involved raising awareness among older adults about the wearing time of the devices. This approach aimed to ensure that individuals were consistently wearing the devices for the recommended duration. The second intervention strategy involved the use of goal-setting tools. By setting specific goals related to wearing the WMDs, older adults were more likely to adhere to the prescribed use. These goal-setting tools could provide individuals with the structure and motivation to incorporate the use of WMDs into their daily routines.

It is surprising that none of the interventions in the included articles were primarily aimed at promoting adherence to WMDs but rather at examining the effectiveness of the WMDs in enhancing health outcomes. For instance, there was a study about the effectiveness of WMDs on increasing step count, but no data were presented on the wearing time of the WMDs [22]. Another study examined adherence to an intervention program [23]. This study focused on assessing how well participants adhered to a specific intervention or treatment program rather than on specifically evaluating adherence to WMDs. Furthermore, most of the cross-sectional studies identified in the systematic review concentrated on investigating the reasons and factors influencing the use or nonuse of WMDs by older adults [5,7,9,24-26]. These studies aimed to understand the barriers and facilitators associated with older adults’ adoption and acceptance of WMDs but did not explore how older adults can sustain the use of WMDs over an extended period.

It is also noteworthy that the pilot study on organ transplant recipients [19] was related to another parent article [18], which theoretically followed up on the same population and intervention at two different time points. The decrease in the WMD adherence rate from 6 months (intervention: 100%; control: 92.3%) to 12 months (intervention: 96.5%; control: 80.8%) implies that interventions targeting adherence should be implemented to enhance the sustained use of WMDs for health monitoring. Previous studies found that the barriers contributing to the nonuse of WMDs included technological concerns and user experiences [25-27]. For instance, a lack of skills to use the WMDs, a feeling of uncomfortableness, and inaccuracies in the physiological data collected by WMDs reduced their use [27]. Additionally, inadequate support from health care providers to encourage the use of WMDs may reduce the patients’ trust in the accuracy of the data, hence discouraging WMD use [24]. Both eliminating such barriers and identifying facilitators are necessary to enhance adherence to WMDs.

Our results from 2 studies [18,19] were consistent with the findings from other studies. For instance, previous research has shown that having health care professionals assist older adults in setting health-related goals and providing them with feedback is a crucial factor influencing adherence to WMDs [11,26]. This is because personalized goals and individualized interventions can motivate older adults to achieve desired health outcomes [11] and empower them to make choices related to their health [28]. For example, the SystemCHANGE approach, as illustrated in the 2 studies [18,19], involved guiding participants to collaboratively establish goals with health care professionals by modifying their current routines, thereby making the goals more attainable.

In addition, O’Brien et al [19] emphasized the importance of consistently following up and providing physical incentives, such as gift cards, to enhance motivation among older adults to sustain the use of WMDs. On the other hand, Lutjeboer et al [20] found that older adults who were aware of being tracked in their footwear use demonstrated improved adherence. This finding can be explained by the Hawthorne effect in psychology, whereby participants may alter their behavior when they are aware of being observed [29]. In the case of orthopedic footwear being prescribed by physicians, older adults may seek to establish a therapeutic relationship by meeting the expectations of their physician. Furthermore, older adults may exhibit a greater willingness to wear the footwear when they are pursuing health-related goals [25].

Concerning whether professional-led or peer-led intervention programs are more effective in enhancing WMD adherence, more information is needed before coming to a conclusion. Combining the results from the included studies, health care professionals were found to have played an important role in assisting the participants in improving WMD adherence. A probable explanation for this is that health care professionals, such as physicians and nurses, could monitor the progress of older adults and answer any questions they might have [30]. However, it is unclear whether the effect of peers is comparable to that of professionals. For example, in O’Brien et al’s studies [18,19], monthly sessions were carried out in groups. This could imply that aside from health care professionals, peer sharing and support may help to enhance the WMD adherence of older adults. However, the report lacks detailed information on the group sessions, making it difficult to compare the effects of professional-led and peer-led intervention programs.

Our study has some limitations. Even though we planned to search for articles published during the past 13 years, the articles that matched our criteria for inclusion were only published in recent years. In addition, a meta-analysis on the topic could not be performed due to the small number of articles and their heterogeneity. This could be because WMDs have only become popular in recent years. Another limitation is linked to population bias. This report involved a small sample size of 154 older adults in total, and the population is assumed to be the same in 2 of the included studies. Meanwhile, all of the included studies targeted Western populations, so the results may not be generalizable to people in other countries because factors such as technological know-how could influence WMD use behavior.

### Conclusions

Our findings revealed that low-cost interventions were effective in increasing adherence to WMDs. Several interventions have been identified as effective in promoting adherence to WMDs. However, it is important to note that further studies are needed to validate the results and assess the effectiveness of these interventions. Researchers should also explore other potential behavior change interventions that could be beneficial in improving adherence to WMDs, with a particular focus on community-dwelling older adults. By expanding the range of interventions and conducting more comprehensive studies, we can enhance our understanding of the most effective strategies for improving adherence to WMDs in this specific population.

## Appendix

Multimedia Appendix 1 Search terms in each database.

Multimedia Appendix 2 PRISMA checklist.

## Abbreviations

CENTRAL: Cochrane Central Register of Controlled Trials
PICO: population, intervention, comparison, and outcomes
PRISMA: Preferred Reporting Items for Systematic Reviews and Meta-Analyses
RCT: randomized controlled trial
RoB 2: Cochrane Risk of Bias tool version 2
WMD: wearable monitoring device

## References

[1] (2022). Ageing and health. World Health Organization.

[2] (2020). Healthy ageing and functional ability. World Health Organization.

[3] Yetisen AK, Martinez-Hurtado JL, Ünal Barış, Khademhosseini A, Butt H (2018). Wearables in medicine. Adv Mater.

[4] Ringeval M, Wagner G, Denford J, Paré G, Kitsiou S (2020). Fitbit-based interventions for healthy lifestyle outcomes: systematic review and meta-analysis. J Med Internet Res.

[5] Ferguson C, Hickman LD, Turkmani S, Breen P, Gargiulo G, Inglis SC (2021). "Wearables only work on patients that wear them": barriers and facilitators to the adoption of wearable cardiac monitoring technologies. Cardiovasc Digit Health J.

[6] Huang Y, Upadhyay U, Dhar E, Kuo L, Syed-Abdul S (2022). A scoping review to assess adherence to and clinical outcomes of wearable devices in the cancer population. Cancers (Basel).

[7] Burford K, Golaszewski NM, Bartholomew J (2021). "I shy away from them because they are very identifiable": a qualitative study exploring user and non-user's perceptions of wearable activity trackers. Digit Health.

[8] Creaser AV, Hall J, Costa S, Bingham DD, Clemes SA (2022). Exploring families' acceptance of wearable activity trackers: a mixed-methods study. Int J Environ Res Public Health.

[9] Lacey A, Whyte E, O'Keeffe Sinéad, O'Connor Siobhán, Moran K (2022). A qualitative examination of the factors affecting the adoption of injury focused wearable technologies in recreational runners. PLoS One.

[10] Lee AYL, Wong AKC, Hung TTM, Yan J, Yang S (2022). Nurse-led telehealth intervention for rehabilitation (telerehabilitation) among community-dwelling patients with chronic diseases: systematic review and meta-analysis. J Med Internet Res.

[11] Peng W, Li L, Kononova A, Cotten S, Kamp K, Bowen M (2021). Habit formation in wearable activity tracker use among older adults: qualitative study. JMIR Mhealth Uhealth.

[12] Wong KC, Wong FKY, Chang KKP (2015). Health-social partnership intervention programme for community-dwelling older adults: a research protocol for a randomized controlled trial. J Adv Nurs.

[13] Wong AKC, Bayuo J, Wong FKY, Chow KKS, Wong SM, Lau ACK (2023). The synergistic effect of nurse proactive phone calls with an mHealth app program on sustaining app usage: 3-arm randomized controlled trial. J Med Internet Res.

[14] Wong AKC, Wong FKY, Chow KKS, Wong SM, Lee PH (2021). Effect of a telecare case management program for older adults who are homebound during the COVID-19 pandemic: a pilot randomized clinical trial. JAMA Netw Open.

[15] Page MJ, McKenzie JE, Bossuyt PM, Boutron I, Hoffmann TC, Mulrow CD, Shamseer L, Tetzlaff JM, Akl EA, Brennan SE, Chou R, Glanville J, Grimshaw JM, Hróbjartsson A, Lalu MM, Li T, Loder EW, Mayo-Wilson E, McDonald S, McGuinness LA, Stewart LA, Thomas J, Tricco AC, Welch VA, Whiting P, Moher D (2021). The PRISMA 2020 statement: an updated guideline for reporting systematic reviews. BMJ.

[16] Sterne JAC, Savović J, Page MJ, Elbers RG, Blencowe NS, Boutron I, Cates CJ, Cheng H, Corbett MS, Eldridge SM, Emberson JR, Hernán Miguel A, Hopewell S, Hróbjartsson Asbjørn, Junqueira DR, Jüni Peter, Kirkham JJ, Lasserson T, Li T, McAleenan A, Reeves BC, Shepperd S, Shrier I, Stewart LA, Tilling K, White IR, Whiting PF, Higgins JPT (2019). RoB 2: a revised tool for assessing risk of bias in randomised trials. BMJ.

[17] Popay J, Roberts H, Sowden A, Petticrew M, Arai L, Rodgers M (2005). Guidance on the conduct of narrative synthesis in systematic reviews. A product from the ESRC Methods Programme. Version 1. JECH.

[18] O'Brien T, Tan A, Rose K, Focht B, Daloul R (2021). Maintenance phase of a physical activity intervention in older kidney transplant recipients: a 12-month follow-up. Geriatr Nurs.

[19] O'Brien T, Russell CL, Tan A, Mion L, Rose K, Focht B, Daloul R, Hathaway D (2020). A pilot randomized controlled trial using SystemCHANGE™ approach to increase physical activity in older kidney transplant recipients. Prog Transplant.

[20] Lutjeboer T, van Netten Jaap J, Postema K, Hijmans J (2020). Effect of awareness of being monitored on wearing of orthopaedic footwear. J Rehabil Med.

[21] Higgins JPT, Thomas J, Chandler J, Cumpston M, Li T, Page MJ, Welch VA Cochrane handbook for systematic reviews of interventions version 6.2 (updated February 2021). Cochrane Training.

[22] Kullgren JT, Harkins KA, Bellamy SL, Gonzales A, Tao Y, Zhu J, Volpp KG, Asch DA, Heisler M, Karlawish J (2014). A mixed-methods randomized controlled trial of financial incentives and peer networks to promote walking among older adults. Health Educ Behav.

[23] Grossman J, Arigo D, Bachman JL (2018). Meaningful weight loss in obese postmenopausal women: a pilot study of high-intensity interval training and wearable technology. Menopause.

[24] Godfrey A, Stuart S (2021). Digital Health: Exploring Use and Integration of Wearables.

[25] Li L, Peng W, Kononova A, Bowen M, Cotten SR (2020). Factors associated with older adults' long-term use of wearable activity trackers. Telemed J E Health.

[26] Moore K, O'Shea E, Kenny L, Barton J, Tedesco S, Sica M, Crowe C, Alamäki Antti, Condell J, Nordström Anna, Timmons S (2021). Older adults' experiences with using wearable devices: qualitative systematic review and meta-synthesis. JMIR Mhealth Uhealth.

[27] Lu L, Zhang J, Xie Y, Gao F, Xu S, Wu X, Ye Z (2020). Wearable health devices in health care: narrative systematic review. JMIR Mhealth Uhealth.

[28] Baker A, Cornwell P, Gustafsson L, Stewart C, Lannin NA (2022). Developing tailored theoretically informed goal-setting interventions for rehabilitation services: a co-design approach. BMC Health Serv Res.

[29] Adair JG (1984). The Hawthorne effect: a reconsideration of the methodological artifact. J Applied Psychol.

[30] Atreja A, Bellam N, Levy SR (2005). Strategies to enhance patient adherence: making it simple. MedGenMed.

