# Supplementary material for: Effects of Peer- or Professional-Led Support in Enhancing Adherence to Wearable Monitoring Devices Among Community-Dwelling Older Adults: Systematic Review of Randomized Controlled Trials
Source: J Med Internet Res. 2024 Jun 20;26:e53607. doi: 10.2196/53607 (PMC11224698; doi:10.2196/53607)
Supplement: Multimedia Appendix 1 [file jmir_v26i1e53607_app1.docx]

**Appendix 1- Search terms in each database**

*Population*

elderl* OR aged OR aging OR ageing OR “aging adult*” OR “ageing adult*” OR “old adult*” OR “older adult*” OR “older person” OR “older people” OR “elderly person” OR senior* OR geriatric

AND

“wearable monitoring device*” OR “health monitoring device*” OR “wearable monitor*” OR “wearable device*” OR “wearable electronic device*” OR “wearable sensor*” OR “wearable monitor*” OR “health track*” OR tracker* OR tracking OR “wearable technolog*” OR “wearable patient monitor*” OR “digital health” OR ehealth OR mhealth OR telemonitor* OR “smart watch*” OR smartwatch* OR “smart wearable*”

AND

*Intervention*

education* OR intervention* OR program*

AND

*Outcome*

adhere* OR non-adhere* OR nonadhere* OR complian* OR non-complian* OR noncomplian* OR comply OR persist* OR refusal OR refuse* OR accept* OR conform* OR engag* OR intent* OR satisf*

**Databases search**

**Cochrane Central Register of Controlled Trials [CENTRAL]**

|  | Query |
| --- | --- |
| #1 | elderl* OR aged OR aging OR ageing OR “aging adult*” OR “ageing adult*” OR “old adult*” OR “older adult*” OR “older person” OR “older people” OR “elderly person” OR senior* OR geriatric |
| #2 | MeSH descriptor: [Aged] in all MeSH products |
| #3 | MeSH descriptor: [Frail Elderly] explode all trees |
| #4 | #1 OR #2 OR #3 |
| #5 | “wearable monitoring device*” OR “health monitoring device*” OR “wearable monitor*” OR “wearable device*” OR “wearable electronic device*” OR “wearable sensor*” OR “wearable monitor*” OR “health track*” OR tracker* OR tracking OR “wearable technolog*” OR “wearable patient monitor*” OR “digital health” OR ehealth OR mhealth OR telemonitor* OR “smart watch*” OR smartwatch* OR “smart wearable*” |
| #6 | MeSH descriptor: [Wearable Electronic Devices] explode all trees |
| #7 | #5 OR #6 |
| #8 | education* OR intervention* OR program* |
| #9 | MeSH descriptor: [Internet-Based Intervention] explode all trees |
| #10 | #8 OR #9 |
| #11 | adhere* OR non-adhere* OR nonadhere* OR complian* OR non-complian* OR noncomplian* OR comply OR persist* OR refusal OR refuse* OR accept* OR conform* OR engag* OR intent* OR satisf* |
| #12 | #4 AND #7 AND #10 AND #11 |
| #13 | #12 with Publication Year from 2010 to present, in Trials |

**PubMed**

|  | Query |
| --- | --- |
| S1 | elderl* OR aged OR aging OR ageing OR “aging adult*” OR “ageing adult*” OR “old adult*” OR “older adult*” OR “older person” OR “older people” OR “elderly person” OR senior* OR geriatric OR “Aged”[Mesh] OR “Frail Elderly”[Mesh] |
| S2 | “wearable monitoring device*” OR “health monitoring device*” OR “wearable monitor*” OR “wearable device*” OR “wearable electronic device*” OR “wearable sensor*” OR “wearable monitor*” OR “health track*” OR tracker* OR tracking OR “wearable technolog*” OR “wearable patient monitor*” OR “digital health” OR ehealth OR mhealth OR telemonitor* OR “smart watch*” OR smartwatch* OR “smart wearable*” OR “wearable electronic devices”[MeSH] |
| S3 | education* OR intervention* OR program* OR “Internet-Based Intervention”[Mesh] OR “Clinical Trial” [Publication Type] |
| S4 | adhere* OR non-adhere* OR nonadhere* OR complian* OR non-complian* OR noncomplian* OR comply OR persist* OR refusal OR refuse* OR accept* OR conform* OR engag* OR intent* OR satisf* |
| S5 | S1 AND S2 AND S3 AND S4 |
| S6 | S5  Filters applied: Randomized Controlled Trial, Humans, Middle Aged + Aged: 45+ years, Middle Aged: 45-64 years, Aged: 65+ years, 80 and over: 80+ years, from 2010/1/1 - 2023/1/26. |

**EMBASE**

|  | Query |
| --- | --- |
| #1 | elderl* OR aged OR aging OR ageing OR “aging adult*” OR “ageing adult*” OR “old adult*” OR “older adult*” OR “older person” OR “older people” OR “elderly person” OR senior* OR geriatric OR “frail elderly” |
| #2 | “wearable monitoring device*” OR “health monitoring device*” OR “wearable monitor*” OR “wearable device*” OR “wearable electronic device*” OR “wearable sensor*” OR “wearable monitor*” OR “health track*” OR tracker* OR tracking OR “wearable technolog*” OR “wearable patient monitor*” OR “digital health” OR ehealth OR mhealth OR telemonitor* OR “smart watch*” OR smartwatch* OR “smart wearable*” |
| #3 | education* OR intervention* OR program* |
| #4 | adhere* OR non-adhere* OR nonadhere* OR complian* OR non-complian* OR noncomplian* OR comply OR persist* OR refusal OR refuse* OR accept* OR conform* OR engag* OR intent* OR satisf* |
| #5 | #1 AND #2 AND #3 AND #4 |
| #6 | #5 AND ([randomized controlled trial]/lim OR “controlled clinical trial”/de) AND [humans]/lim AND [2020-2023]/py |

**PsycINFO**

|  | Query |
| --- | --- |
| #1 | elderl* OR aged OR aging OR ageing OR “aging adult*” OR “ageing adult*” OR “old adult*” OR “older adult*” OR “older person” OR “older people” OR “elderly person” OR senior* OR geriatric OR aged OR “frail elderly” |
| #2 | “wearable monitoring device*” OR “health monitoring device*” OR “wearable monitor*” OR “wearable device*” OR “wearable electronic device*” OR “wearable sensor*” OR “wearable monitor*” OR “health track*” OR tracker* OR tracking OR “wearable technolog*” OR “wearable patient monitor*” OR “digital health” OR ehealth OR mhealth OR telemonitor* OR “smart watch*” OR smartwatch* OR “smart wearable*” |
| #3 | education* OR intervention* OR program* |
| #4 | adhere* OR non-adhere* OR nonadhere* OR complian* OR non-complian* OR noncomplian* OR comply OR persist* OR refusal OR refuse* OR accept* OR conform* OR engag* OR intent* OR satisf* |
| #5 | #1 AND #2 AND #3 AND #4 |
| #6 | #5  [Applied filters: 2010-01-01 - 2023-01-26; NOT (Dissertation Abstract AND Book AND Edited Book AND Chapter AND Handbook/Manual AND Erratum/Correction AND Letter AND Comment/Reply AND Column/Opinion); NOT (Inpatient AND Animal)] |

**British Nursing Index**

|  | Query |
| --- | --- |
| #1 | elderl* OR aged OR aging OR ageing OR “aging adult*” OR “ageing adult*” OR “old adult*” OR “older adult*” OR “older person” OR “older people” OR “elderly person” OR senior* OR geriatric OR aged OR “frail elderly” |
| #2 | “wearable monitoring device*” OR “health monitoring device*” OR “wearable monitor*” OR “wearable device*” OR “wearable electronic device*” OR “wearable sensor*” OR “wearable monitor*” OR “health track*” OR tracker* OR tracking OR “wearable technolog*” OR “wearable patient monitor*” OR “digital health” OR ehealth OR mhealth OR telemonitor* OR “smart watch*” OR smartwatch* OR “smart wearable*” |
| #3 | education* OR intervention* OR program* |
| #4 | adhere* OR non-adhere* OR nonadhere* OR complian* OR non-complian* OR noncomplian* OR comply OR persist* OR refusal OR refuse* OR accept* OR conform* OR engag* OR intent* OR satisf* |
| #5 | #1 AND #2 AND #3 AND #4 |
| #6 | #5 AND Filters:  2010-01-01 - 2023-01-26  NOT (General Information AND Commentary AND Editorial AND News AND Literature Review AND Case Study AND Conference Proceeding AND Review AND Front Page/Cover Story AND Correspondence AND Instructional Material/Guideline AND Biography AND Conference AND Front Matter AND Interview AND Letter To The Editor AND Obituary AND Table Of Contents)  NOT (mortality AND nursing education AND systematic review AND children & youth AND dementia AND pediatrics AND students AND interviews AND teenagers AND pregnancy AND children)  ((elderl* OR aged OR aging OR ageing OR “aging adult*” OR “ageing adult*” OR “old adult*” OR “older adult*” OR “older person” OR “older people” OR “elderly person” OR senior* OR geriatric OR aged OR “frail elderly”) AND (“wearable monitoring device*” OR “health monitoring device*” OR “wearable monitor*” OR “wearable device*” OR “wearable electronic device*” OR “wearable sensor*” OR “wearable monitor*” OR “health track*” OR tracker* OR tracking OR “wearable technolog*” OR “wearable patient monitor*” OR “digital health” OR ehealth OR mhealth OR telemonitor* OR “smart watch*” OR smartwatch* OR “smart wearable*”) AND (education* OR intervention* OR program*) AND (adhere* OR non-adhere* OR nonadhere* OR complian* OR non-complian* OR noncomplian* OR comply OR persist* OR refusal OR refuse* OR accept* OR conform* OR engag* OR intent* OR satisf*)) NOT (at.exact(“General Information” OR “Commentary” OR “Editorial” OR “News” OR “Literature Review” OR “Case Study” OR “Conference Proceeding” OR “Review” OR “Front Page/Cover Story” OR “Correspondence” OR “Instructional Material/Guideline” OR “Biography” OR “Conference” OR “Front Matter” OR “Interview” OR “Letter to the Editor” OR “Obituary” OR “Table Of Contents”) NOT subt.exact(“mortality” OR “nursing education” OR “systematic review” OR “children & youth” OR “dementia” OR “pediatrics” OR “students” OR “interviews” OR “teenagers” OR “pregnancy” OR “children”) AND pd(20100101-20230126)) |

**Web of Science**

|  | Query |
| --- | --- |
| #1 | elderl* OR aged OR aging OR ageing OR “aging adult*” OR “ageing adult*” OR “old adult*” OR “older adult*” OR “older person” OR “older people” OR “elderly person” OR senior* OR geriatric OR aged OR “frail elderly” |
| #2 | “wearable monitoring device*” OR “health monitoring device*” OR “wearable monitor*” OR “wearable device*” OR “wearable electronic device*” OR “wearable sensor*” OR “wearable monitor*” OR “health track*” OR tracker* OR tracking OR “wearable technolog*” OR “wearable patient monitor*” OR “digital health” OR ehealth OR mhealth OR telemonitor* OR “smart watch*” OR smartwatch* OR “smart wearable*” |
| #3 | education* OR intervention* OR program* |
| #4 | adhere* OR non-adhere* OR nonadhere* OR complian* OR non-complian* OR noncomplian* OR comply OR persist* OR refusal OR refuse* OR accept* OR conform* OR engag* OR intent* OR satisf* |
| #5 | #1 AND #2 AND #3 AND #4 |
| #6 | #5 AND Filters  #4 AND #3 AND #2 AND #1 and 2010 or 2011 or 2012 or 2013 or 2014 or 2015 or 2016 or 2017 or 2018 or 2019 or 2020 or 2021 or 2022 or 2023 (Publication Years) and Review Article or Proceeding Paper or Editorial Material or Meeting Abstract or Letter or News Item (Exclude – Document Types) and Agriculture or Energy Fuels or Pediatrics or Environmental Sciences Ecology or Surgery or Chemistry or Business Economics or Geology or Radiology Nuclear Medicine Medical Imaging or Materials Science or Physics or Information Science Library Science or Genetics Heredity or Tropical Medicine or Family Studies or Astronomy Astrophysics or Zoology or Parasitology or Cell Biology or Virology or Biochemistry Molecular Biology or Physical Geography or Geography or Transportation or Geochemistry Geophysics or Sociology or Marine Freshwater Biology or Mathematical Computational Biology or Water Resources or Biodiversity Conservation or Biophysics or Construction Building Technology or Emergency Medicine or Government Law or Oceanography or Evolutionary Biology or Forestry or Fisheries or Meteorology Atmospheric Sciences or Paleontology or Anthropology or Mathematics or Pathology or Urban Studies or Criminology Penology or Development Studies or Developmental Biology or Electrochemistry or Plant Sciences or Polymer Science or Toxicology or Veterinary Sciences or Acoustics or Anatomy Morphology or History or Imaging Science Photographic Technology or Religion or Architecture or Area Studies or Art or Dance or Entomology or History Philosophy Of Science or Mathematical Methods In Social Sciences or Music or Philosophy or Theater or Metallurgy Metallurgical Engineering or Microbiology (Exclude – Research Areas) |

**CINAHL**

|  | Query |
| --- | --- |
| #1 | elderl* OR aged OR aging OR ageing OR “aging adult*” OR “ageing adult*” OR “old adult*” OR “older adult*” OR “older person” OR “older people” OR “elderly person” OR senior* OR geriatric OR aged OR “frail elderly” |
| #2 | “wearable monitoring device*” OR “health monitoring device*” OR “wearable monitor*” OR “wearable device*” OR “wearable electronic device*” OR “wearable sensor*” OR “wearable monitor*” OR “health track*” OR tracker* OR tracking OR “wearable technolog*” OR “wearable patient monitor*” OR “digital health” OR ehealth OR mhealth OR telemonitor* OR “smart watch*” OR smartwatch* OR “smart wearable*” |
| #3 | education* OR intervention* OR program* |
| #4 | adhere* OR non-adhere* OR nonadhere* OR complian* OR non-complian* OR noncomplian* OR comply OR persist* OR refusal OR refuse* OR accept* OR conform* OR engag* OR intent* OR satisf* |
| #5 | #1 AND #2 AND #3 AND #4 |
| #6 | #5 [Limiters - Published Date: 20100101-; Randomized Controlled Trials] |
